# Supplementary material for: Hair-Based Assessment of Sex Steroid Hormones in Patients with Anorexia Nervosa
Source: Metabolites. 2022 Dec 22;13(1):21. doi: 10.3390/metabo13010021 (PMC9863132; doi:10.3390/metabo13010021)
Supplement: Supplementary file 1 [file metabolites-13-00021-s001.zip › metabolites-2058159-supplementary.pdf]

Supplemental Materials

Hair-based assessment of sex steroid hormones in patients with anorexia nervosa

SM 1 Methods

SM 1.1 Study participants

Participants of all groups were excluded if they had a history of any of the following diagnoses: organic brain syndrome, schizophrenia, substance dependence, psychosis not otherwise specified, bipolar disorder, bulimia nervosa, or binge-eating disorder. Further exclusion criteria for all participants were an IQ below 85, current substance abuse, inflammatory, neurologic, or metabolic illness, chronic medical or neurological illness that could affect appetite, eating behavior or body weight, clinically relevant anemia, pregnancy, or breast feeding.

SM 1.2 Participant medication history

No participant was on psychotropic medication within 4 weeks prior to study participation (cross-sectional study time point: AN-T1 and HC). In our longitudinal sample (AN-T1/T2), 2 AN patients were on psychotropic medication: 1 patient took a selective serotonin reuptake inhibitor between day 0 and 30 (after treatment initiation), and 1 patient took mirtazapine between day 30 and 60 (after treatment initiation).

SM 1.3 Hormone measurements in blood plasma (leptin, ghrelin)

To measure leptin and ghrelin levels, venous blood was collected into vacutainer tubes between 7 and 9 a.m. after an overnight fast. EDTA-plasma samples (aprotinin was added, activity 270 KIU per ml) were centrifuged (2800xg for 15 min), aliquoted, and stored at -80°C until analysis. Plasma concentrations of leptin/ghrelin in all participants were measured with a commercially available enzyme-linked immunosorbent assay at the Institute for Clinical Chemistry and Pathobiochemistry, Otto-von-Guericke-University Magdeburg, according to the manufacturer's instructions (BioVendor; Czech Republic) with an intra-assay coefficient of variation (CV) of 4.2 %, inter-assay CV of 6.7 %, and a lower limit of detection of 0.2 ng/ml.

SM 2 Results

SM Table S1. Frequency of observations for progesterone and DHEA below the lowest detectable value (defined as the limit of detection, LOD) for each group in the cross-sectional sample, sorted by age.

	Progesterone		DHEA	
Age Range	AN<br>N = 18	HC<br>N = 7	AN<br>N = 6	HC<br>N = 2
12	3	3	1	1
13	5	2	2	1
14	5	1	0	0
15	2	0	2	0
16	2	1	0	0
17	1	0	1	0

AN = acute anorexia nervosa; HC = healthy control; DHEA = Dehydroandrosterone.

SM 2.1 Chi-Squared tests in the cross-sectional study sample

Closer examination of hair progesterone levels below vs. above LOD revealed large differences between the AN (54,5 %) and HC group (21,2 %). For hair DHEA, values below LOD were 18,2 % in the AN and 6,1 % in the HC group. Chi-squared test of independence showed that there was a significant association in hair progesterone (X^2 (1, N = 66) = 7.79, p = .005) but not in hair DHEA (X^2 (1, N = 66) = 2.28, p = .131) between values below vs. above LOD and participant group.

SM 2.2 Friedman tests in the longitudinal study sample

In our longitudinal AN sample, there was no significant difference between the 4 time points (T1, $T2_{1\text{-}3}$) in hair progesterone and DHEA concentrations. For hair progesterone, the non-parametric Friedman test of differences among repeated measures yielded a Chi-squared value of 1.50 which was not significant (p = .682). A further analysis for hair DHEA revealed a Chi-squared value of 2.32 which was also not significant (p = .509).

SM 2.3 Chi-Squared tests in the longitudinal study sample

An examination of all 4 time periods showed that about 60% (58.62 – 65.52 %) of all values were below LOD in hair progesterone and about 10% (10.34 – 13.79 %) in hair DHEA. Chi-squared tests of independence showed that there was no significant association in hair progesterone (X^2 (3, $N = 116$) = .40, $p = .94$) or hair DHEA (X^2 (3, $N = 116$) = .23, $p = .97$) between values below vs. above LOD and time periods.

SM 2.4 Hair hormone measures in AN-$T2_3$ compared to HC

Mann-Whitney-U-Tests revealed there was still a significantly lower hair progesterone level in the AN-$T2_3$ group ($N = 29$) compared to HC ($N = 33$; $U = 737.50$, $p < .001$), and still no differences in hair DHEA ($U = 512.00$, $p = .636$).

SM 2.5 Post hoc power analyses

We conducted post-hoc power analyses that showed a power (1 – ß error probability) of 0.98 for group differences of progesterone levels, 0.30 for DHEA levels and for our longitudinal sample (progesterone, T1 – $T2_3$) a power (1 – ß error probability) of 0.90.

References

1. Gao, W.; Stalder, T.; Foley, P.; Rauh, M.; Deng, H.; Kirschbaum, C. Quantitative Analysis of Steroid Hormones in Human Hair Using a Column-Switching LC–APCI–MS/MS Assay. *J. Chromatogr. B* **2013**, *928*, 1–8, doi:10.1016/j.jchromb.2013.03.008.
